# Increased frequency of canine distemper virus‐specific antibodies in multiple sclerosis

**DOI:** 10.1002/brb3.1920

**Published:** 2020-12-10

**Authors:** Christine Rohowsky‐Kochan, Amy Davidow, Peter Dowling, Stuart D. Cook

**Affiliations:** ^1^ Department of Pharmacology, Physiology and Neuroscience Rutgers, The State University of New Jersey New Jersey Medical School Newark NJ USA; ^2^ Department of Biostatistics & Epidemiology Division of Biostatistics Rutgers School of Public Health Newark NJ USA; ^3^ Veterans Administration Medical Center Neurology Service East Orange NJ USA; ^4^ Department of Neurology Rutgers, The State University of New Jersey New Jersey Medical School Newark NJ USA

**Keywords:** antibodies, canine distemper virus, multiple sclerosis, peptide ELISA

## Abstract

**Background and purpose:**

Canine distemper virus (CDV) is a candidate agent in the etiology of multiple sclerosis (MS). Elevated anti‐CDV levels were previously found in the sera from MS patients compared with controls. We now investigated whether there was an age‐related association with the presence of antibodies specific to CDV‐hemagglutinin (H) protein in MS.

**Methods:**

Sera from patients with MS, other neurological diseases, and inflammatory and/or autoimmune diseases, and healthy individuals were screened for anti‐CDV in an ELISA using linear peptides of the CDV‐H protein as antigen. Antibody levels to measles and *varicella‐zoster virus* were measured and served as controls.

**Results:**

Analysis of the new cohort of MS patients and controls confirmed our initial finding of elevated anti‐CDV‐H levels in MS patients. An increase in measles but not *varicella‐zoster virus* antibody levels was found in MS patients compared with healthy controls. Data from the new cohort of patients and controls were combined with data from the original study and analyzed with respect to age distribution of anti‐CDV IgG. Mean CDV antibody levels were significantly elevated in each decade from 20 to 50 years of age in MS compared with healthy and disease controls. Antibody levels to measles virus were not consistently elevated during this age span. A striking relationship (*p* < .0001, odds ratio = 5.0) was observed between elevated anti‐CDV‐H levels and diagnosis of MS.

**Conclusions:**

The finding that anti‐CDV levels are elevated in MS patients of all ages provides substantial evidence of a strong association between elevated anti‐CDV and MS.

## Graphical abstract

We provide evidence that canine distemper virus is associated with patients with multiple sclerosis. Antibodies to this virus were found in multiple sclerosis patients throughout many decades.

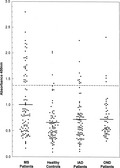

## 1 INTRODUCTION

Multiple sclerosis (MS), a demyelinating disease of the central nervous system (CNS), is believed to have an infectious etiology although evidence supporting this is indirect. We proposed that in some instances, MS may be due to a zoonotic infection and that the morbillivirus, canine distemper virus (CDV), is a possible candidate in the causation of MS. Epidemiologic studies (Cook et al., 1978, 1979, 1987; Cook & Dowling, 1982; Lincoln et al., 2008) and experimental animal models (Higgins et al., 1989; Vandevelde et al., 1982) of viral‐induced demyelination provide circumstantial evidence in support of the CDV‐MS hypothesis.

Serological studies searching for CDV‐specific antibodies (Abs) were hampered due to the high degree of homology between CDV and the closely related measles virus (MV). We devised a peptide‐based enzyme‐linked immunoabsorbent assay (P­ELISA) using peptide sequences predicted to contain CDV‐specific antigenic determinants to search for CDV Abs in animal and human sera (Rohowsky‐Kochan et al., 1995). Initial studies demonstrated that sera from CDV‐immunized animals reacted with the CDV peptides in P‐ELISA, whereas sera from both animals and humans infected with MV did not react with the CDV peptides (Rohowsky‐Kochan et al., 1995). A threefold increase in CDV antibody titers was observed in MS patients compared to both patients with other neurological disorders and healthy controls. These results provided the first unconfounded serological evidence that MS patients have elevated anti‐CDV levels in support of the hypothesis that MS, in some instances, may be triggered by this neurotropic dog virus.

We now investigated whether there was an age‐related association with the presence of CDV‐H Abs in MS. To perform this analysis, we measured CDV­H Abs in a new (previously not tested) cohort of patients with MS, inflammatory and/or autoimmune disorders (IAD), and other neurological diseases (OND) in order to increase the sample size for each age group. We determined antibody levels to an analogous peptide from the MV‐H protein and to *varicella‐zoster virus* (VZV). We show that anti‐CDV‐H levels were significantly higher in each decade from 20 to 50 years of age in MS patients compared with healthy and disease controls. This study extends and confirms our initial report that the highest levels of anti‐CDV‐H are found in MS patients.

## 2 METHODS

### 2.1 Selection and synthesis of CDV and MV peptides

CDV‐H l, CDV‐H2, and CDV‐H3 peptides correspond to the most hydrophilic and presumed immunogenic regions of the CDV‐H protein as assessed by the Hopp and Woods algorithm (Hopp & Woods, 1981). The sequence of the CDV peptides is as follows: CDV‐H l (residues 234–248)—LVPDDIEREFDTREI; CDV‐H2 (residues 365–380)—ALASEKQEEKGCLES; and CDV‐H3 (residues 70–84)—FSRLLKEDMEKSEAV. There were no structural relationships between the three CDV‐H peptides. A control peptide (16 amino acids in length) corresponding to a predicted antigenic determinant of the MV‐H protein (residues 369–384) was also selected using this algorithm. The sequence of MVH2 is TTRTDDKLRMETCFQQ. MV‐H2 has only a single amino acid identity with its homologous CDV‐H2 peptide. All four peptides were synthesized by Peptides International, Inc., Louisville, KY. The three CDV‐H peptides were >95% pure, while the MV‐H2 peptide was >80% pure as determined by analytical high‐pressure liquid chromatography.

### 2.2 Subjects

Research protocols were approved by the Institutional Review Board in accordance with regulations mandated by the Department of Health and Human Services and the Declaration of Helsinki. Informed consent was obtained from each subject. Sera were obtained from 94 randomly selected patients (mean age was 37.5 years; 61 females: 33 males) with clinically definite MS (Poser et al., 1983). The MS patients had relapsing‐remitting or secondary progressive disease. The demographics of the patients and controls in the present study were similar to those reported in the original study (Rohowsky‐Kochan et al., 1995). The mean duration of MS for 73 patients was 8.8 yr. (range: 1–41 yr.); 13 patients were recently diagnosed (<10 months), whereas in 8 patients the duration of disease was not certain. At the time of blood sampling, 60 MS patients were not on any immunomodulatory drugs, 14 patients were taking oral corticosteroids (mean dosage: 61 mg, range: 10–100 mg), four patients were on oral corticosteroids and imuran (mean dosage: 21 and 150 mg, respectively), two patients were on imuran only (mean dosage: 163 mg), and 5 patients were being treated with interferon‐β (Betaseron^®^). It was not possible to obtain information regarding therapy on nine MS patients.

Sera were obtained from 79 patients (mean age was 44.4 years; 25 females: 54 males) with IAD. The IAD group consisted of patients with inclusion body myositis (20), human immunodeficiency virus‐1 (HIV‐1) infection (16), Guillain–Barre syndrome (13), postpolio syndrome (10), insulin‐dependent diabetes mellitus (5), viral encephalitis (3), inflammatory neuropathies (3) or arthritis (2), Hashimoto's disease (1), temporal arteritis (1), chronic idiopathic polyneuropathy (1), systemic lupus erythematosus (1), idiopathic optic neuritis (1), myasthenia gravis (1), and synovial sarcoma (1). Fifty‐two patients (mean age was 36.4 years; 29 females: 23 males) were included who had OND consisting of amyotrophic lateral sclerosis (29), neuropathy (7), stroke (7), vertigo (2), degenerative disk diseases causing myelopathy and radiculopathy (2), Alzheimer's disease (1), seizure disorder (1), migraine (1), olivopontocerebellar atrophy (1), and hysterical hemiparesis (1). Sera were obtained from 77 healthy controls (HC) (mean age was 36.1 years; 45 females: 32 males) with no known medical or neurological illness. To avoid biased sampling, no information regarding dog or distemper exposure was obtained at the time of blood drawing. Samples were collected over 9 years, handled identically, and stored at −70°C until testing. None of the MS patients or disease and healthy controls had been previously tested for levels of anti‐CDV‐H.

### 2.3 Detection of viral antibodies in human sera

CDV and MV peptide‐specific Abs in sera were detected by P‐ELISA as described (Rohowsky‐Kochan et al., 1995). Briefly, 96‐well U‐bottom microtiter plates were coated overnight with CDV‐H1, CDV‐H2, CDV‐H3, or MV‐H2 peptides, washed, and nonspecific sites were blocked by incubating with 2% fetal bovine serum for 1 hr. Sera (1:10 dilution) were added and incubated for 2 hr at 23°C, plates were washed, and an anti‐human IgG horseradish peroxidase conjugate was added. After 1‐hr incubation in the dark, a substrate solution—2, 2‐azino‐di (3‐ethylbenzthiazoline sulfonic acid) (ABTS)—was added, followed by a brief incubation in the dark, and termination of the reaction with 1% sodium dodecyl sulfate (SDS). Absorbance values (405 nm) were read blindly on an automated microplate reader (BioTek Instruments) and corrected by subtracting optical densities from wells with 1% bovine serum albumin (BSA). Each serum was tested twice in separate assays at randomly assigned positions in the tray. The average of the two absorbance values was used. To control for assay variability, a serum with a high titer of anti‐CDV‐H and a nonreactive serum were tested in each assay. The assay was performed blindly with no biased selection of MS patient samples. Equal numbers of MS and control sera were assayed simultaneously. No diagnosis was written on the test tube or protocol sheet. A serum was considered positive for CDV or MV peptide antibodies if its absorbance value was two SDs above the mean absorbance value for HC. VZV‐specific antibodies in human sera were measured using a commercially available ELISA directed against a varicella‐zoster virus antigen (Wampole Laboratories) as per the manufacturer's instructions and processed similar to the P‐ELISA.

### 2.4 Determination of total serum IgG levels

Microtiter trays were coated with rabbit anti‐human IgG antibody (25 ug/ml) (Jackson ImmunoResearch) in 0.05M carbonate buffer, pH‐9.6, for 48 hr at 4°C. Trays were blocked for 24 hr at 4°C with 1.0% dry milk/phosphate‐buffered saline and dilutions of human IgG standards (Chemicon) or test sera were plated in triplicate and incubated for 2 hr. After washing, a rabbit anti‐human‐lgG horseradish peroxidase conjugate (1:3000, Jackson ImmunoResearch) was added for 1 hr. The reactions were visualized by the addition of ABTS followed by 1% SDS to stop the reaction. Absorbance at 405nm was read on a microplate reader. IgG concentrations were determined by extrapolation from the standard curve plot.

### 2.5 Purification of IgG from human sera

IgG was isolated from human sera using the Affi‐Gel^®^ ID Protein A MAPS II Kit (Bio‐Rad) as per manufacturer's instructions. Briefly, sera were diluted 1:1 with binding buffer and loaded on a protein A‐bound gel column. The column was washed with 10‐bed volumes of binding buffer till the serum protein peak was eluted, and the IgG was eluted using 5‐ to 7‐bed volumes of elution buffer. IgG‐containing fractions were pooled, dialyzed overnight at 4°C with distilled H_2_0, lyophilized, reconstituted with phosphate‐buffered saline, and stored at −70°C.

### 2.6 Statistical analysis

Nonparametric rank‐sum (Kruskal–Wallis and Wilcoxon) tests were performed to compare the distributions of absorbance values of CDV, MV, or VZV Abs in MS, IAD, and OND patients as well as HC. The criterion for significance was two‐tailed *p* < .05, adjusted for multiple comparisons by Bonferroni's inequality. Correlation between the three CDV peptide antibodies and MV antibodies was determined by Pearson's correlation test. The degree of association between positivity for anti‐CDV‐H and MS diagnosis was expressed as an odds ratio (OR) and evaluated by Fisher's exact test.

## 3 RESULTS

### 3.1 Levels of CDV‐H and MV‐H peptide antibodies

**FIGURE 1:**
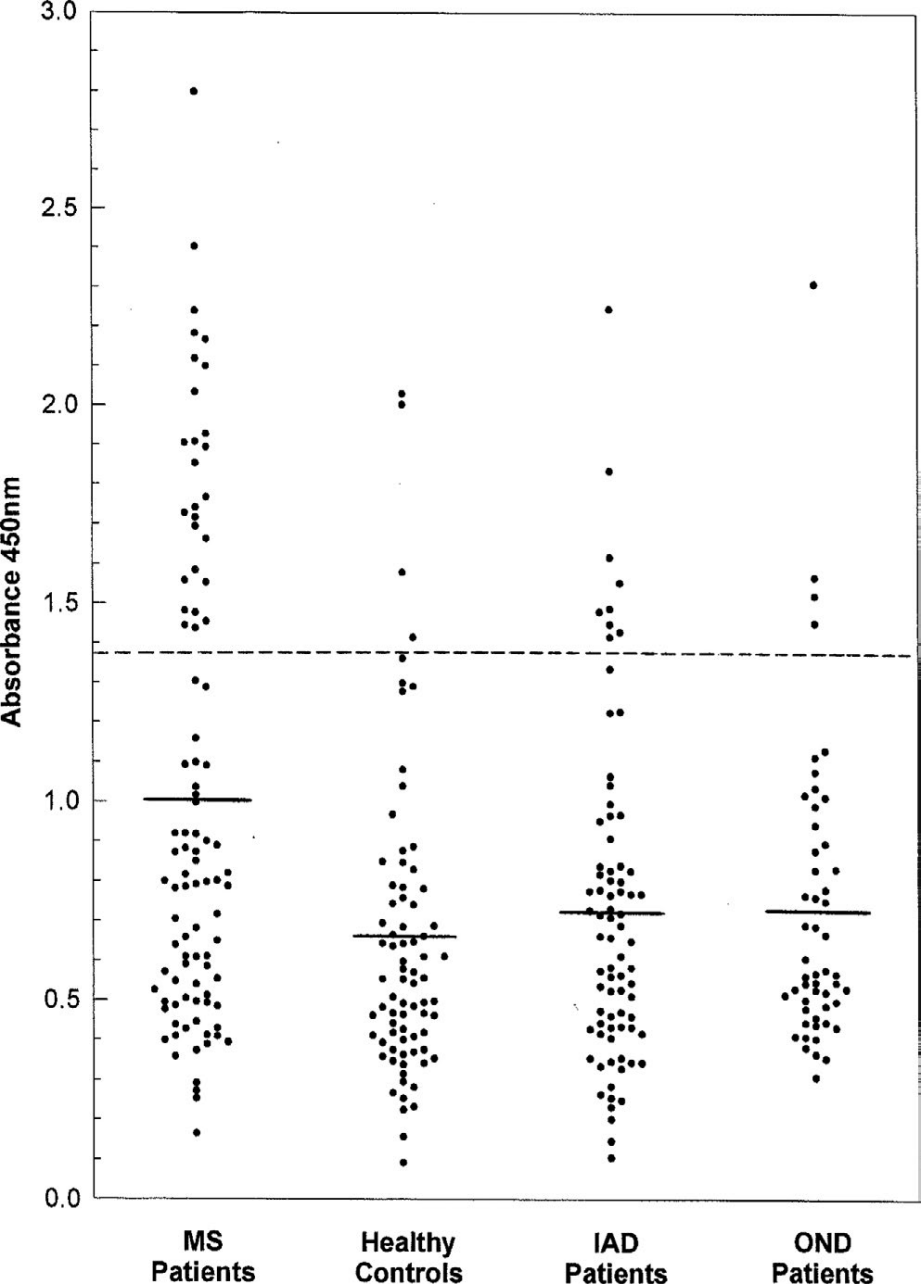
IgG antibodies to the CDV‐Hl peptide in the sera of MS patients and controls. Absorbance values (405 nm) of anti‐CDV‐H1 IgG in patients with MS, IAD, OND, and HC are depicted. Results are expressed as the mean A405 for triplicate determinations obtained in two different assays. Short solid line in each column denotes mean absorbance values of respective group. Absorbance values above 1.34 (long dashed line 2 *SD* above mean A405 value of healthy individuals) were considered positive

We first measured anti‐CDV‐H peptide levels in the new cohort of MS patients, IAD and OND patients, and HC. A significant difference (*p* < .0002) was observed in the absorbance values for anti‐CDV‐H1 among patients with MS, IAD, OND, and HC. Anti‐CDV‐H1 levels were significantly elevated in MS patients (mean ± *SD*, 1.00 ± 0.60) compared with HC (0.65 ± 0.37, *p* < .0001), IAD patients (0.72 ± 0.41, *p* < .0023), and OND patients (0.72 ± 0.37, *p* < .012) (Figure 1). Twenty‐seven of 94 (29%) MS patients had elevated anti‐CDV‐H1 levels as compared with 9 of 79 (11%) IAD patients, 4 of 52 (8%) OND patients, and 4 of 77 (5%) HC.

A significant difference was observed in the levels of anti‐CDV‐H2 (*p* < .004) and anti‐CDV‐H3 (*p* < .002) among patients with MS, IAD, OND, and HC (Table 1). Significantly elevated levels of anti‐CDV‐H2 were found in MS patients compared with HC, IAD, and OND patients. CDV‐H3 levels were significantly elevated in MS patients compared with IAD patients and tended (*p* = .06) to be higher compared with both OND and HC. Twenty‐two percent of MS patients had elevated CDV‐H2 Abs compared with 2% of IAD patients, and 4% of OND patients and HC, whereas 19% of MS patients had elevated CDV‐H3 Abs compared with 2% of IAD patients, 4% of OND patients, and 5% of HC.

**TABLE 1:** ELISA absorbance values of anti‐CDV‐H2, CDV‐H3, and MV‐H2 IgG

Group	Mean absorbance (405 nm) ± standard deviation		
	CDV‐H2	CDV‐H3	MV‐H2
MS	0.92 ± 0.61 (*n* = 94)^a^, ^b^, ^c^, ^d^	0.81 ± 0.57 (*n* = 94)^e^	0.97 ± 0.49 (*n* = 87)^f^, ^g^
HC	0.67 ± 0.35 (*n* = 72)	0.60 ± 0.34 (*n* = 72)	0.78 ± 0.39 (*n* = 71)
IAD	0.58 ± 0.33 (*n* = 45)	0.47 ± 0.32 (*n* = 45)	0.90 ± 0.42 (*n* = 63)
OND	0.65 ± 0.40 (*n* = 45)	0.59 ± 0.35 (*n* = 45)	0.76 ± 0.35 (*n* = 47)

^a^ Number of sera tested against each peptide.

^b^ *p* < .019 in comparison with HC.

^c^ *p* < .001 in comparison with IAD patients.

^d^ *p* < .03 in comparison with OND patients.

^e^ *p* < .0004 in comparison with IAD patients.

^f^ *p* < .005 in comparison with HC.

^g^ *p* < .013 in comparison with OND patients.

A significant difference (*p* < .011) was seen in the levels of MV‐H2 Abs among patients with MS, IAD, OND, and HC. MS patients exhibited significantly higher levels of anti‐MV‐H2 than HC and OND patients but not IAD patients (Table 1). Fifteen percent of MS patients had higher anti‐MV‐H2 levels compared with 5% and 2% of healthy and OND controls, respectively.

### 3.2 Levels of VZV antibodies

There were no significant differences in the levels of anti‐VZV between MS patients (1.208 ± 0.32, *n* = 57) and HC (1.311 ± 0.30, *n* = 34). Similar levels of VZV‐specific Abs were noted in MS patients and HC who had elevated levels of anti‐CDV‐H and those who were negative for distemper antibodies. Levels of VZV‐specific Abs were similar in the IAD and OND controls tested (data not shown).

### 3.3 Association between CDV‐H antibodies and MS diagnosis

We examined the relationship between the presence of elevated anti‐CDV‐H and MS diagnosis. Separate contingency table analyses were performed for the original and present studies. Likewise, comparisons were made between MS patients versus. HC and between MS patients and all controls (HC and other disease patients). In the original study, a significant association (*p* = .001, OR = 6.1, 95% confidence interval [CI] = [l.8, 26.8]) was observed between elevated anti‐CDV‐H levels and MS diagnosis. An OR of 6.0 (95% CI = [2.2, 17.3], *p* = .0002) was observed when patients with other diseases were included. Strikingly similar results were obtained in the present study for the association between elevated levels of CDV‐H Abs and a MS diagnosis (*p* < .0001, OR = 7.4, 95% CI = [2.3, 30.1]). Inclusion of patients with other diseases yielded an OR of 4.5 (95% CI = [2.2, 9.4], *p* < .0001). Analysis of the combined studies showed a highly significant relationship (OR of 5.0, 95% CI = [1.2, 21.5], *p* < .0001) between the presence of elevated anti‐CDV (to all three peptides) and MS diagnosis. After controlling for anti‐MV, the OR increased to 5.6 (95% CI = [1.3, 24.7]), although the increase was not significantly different than the value of 5.0. Restricting the analysis to CDV‐H1 antibodies only, the OR of 7.0 (95% CI = [3.0, 16.3]) was obtained; after controlling for anti‐MV, the OR increased to 8.1 (95% CI = [3.1, 20.9]). In comparison, an OR of 2.9 (95% confidence interval [1.3, 6.9], *p* < .008) was observed between elevated anti‐MV levels and MS diagnosis. In sum, there is an increased risk of MS (OR >5) when antibodies to all three CDV peptides are elevated (higher than 2 *SD* over the distribution in the healthy individuals) and an even greater risk (OR >7) when antibodies to CDV‐H1 are elevated, and this risk is independent of the effect of the measles virus. There was no correlation between anti‐CDV‐H levels and disease duration, age at MS onset, age, or sex.

### 3.4 Combined results from two independent studies

Similar levels of significance and degree of associations observed in the present and original studies justified combining and reanalyzing the data. A homogeneity *p* value of .77 and .83 (with and without OND controls, respectively) for the comparison of the odds ratio of the two studies provided a high degree of confidence that pooling the data was an appropriate analysis choice. For this analysis, IAD patients were grouped together with the OND patients and will be referred to as patients with other diseases (OD). A significant difference (*p* < .0001) was observed in mean antibody levels to each of the three CDV peptides among MS patients, OD patients, and HC. CDV‐H1, CDV‐H2, and CDV‐H3 levels in MS patients were significantly increased compared with HC or OD patients (Table 2). CDV‐H l, CDV‐H2, and CDV‐H3 IgG levels were elevated in 27%, 25%, and 21% of MS patients, respectively, 6%, 4%, and 5% of HC and 8%, 4%, and 5% of OD patients. Antibodies to the MV‐H2 peptide were elevated in 23% of MS patients, 21% of HC, and 17% of OD patients. The majority of positive patient and control sera reacted with all three CDV peptides, although sera that reacted with one or two out of three CDV peptides were identified. There are strong correlations (*p* > .0001) among the CDV antibodies within each patient and control group. R values >0.82 were observed in MS patients (range: 0.93–0.96), HC (range: 0.82–0.91), and OD patients (range: 0.84–0.9) between anti‐CDV‐H1, CDV‐H2, and CDV‐H3. The correlation between anti‐CDV‐H and anti‐MV‐H2 was weaker but still significant. Regressing MV on the three CDV epitopes, all were positively associated with MV in healthy individuals, but among MS individuals, the correlation between MV‐H2 and CDV‐H2 is negative, controlling for the other two CDV epitopes. R values >0.5 were observed in MS patients (range: 0.68–0.74), HC (range: 0.52–0.6), and OD patients (range: 0.5–0.63) between the anti‐CDV‐H and MV‐H2.

**TABLE 2:** Combined ELISA results from two independent studies

Group	Mean absorbance (405) ± standard deviation			
	CDV‐H1	CDV‐H2	CDV‐H3	MV‐H2
MS	1.0 ± 0.57 (*n* = 149)^a^, ^b^	0.95 ± 0.58 (*n* = 149)^b^	0.83 ± 0.56 (*n* = 149)^b^	0.96 ± 0.4 (*n* = 140)
HC	0.64 ± 0.35 (*n* = 141)	0.62 ± 0.32 (*n* = 136)	0.53 ± 0.31 (*n* = 136)	0.87 ± 0.21 (*n* = 126)
OD	0.70 ± 0.38 (*n* = 192)	0.66 ± 0.38 (*n* = 151)	0.57 ± 0.34 (*n* = 151)	0.84 ± 0.39 (*n* = 158)

^a^ Number of sera tested.

^b^ *p* < .0001 in comparison with healthy controls and other disease groups for all three CDV‐H peptides and one MV‐H peptide.

### 3.5 Analysis of CDV‐H antibodies in different age groups

**FIGURE 2:**
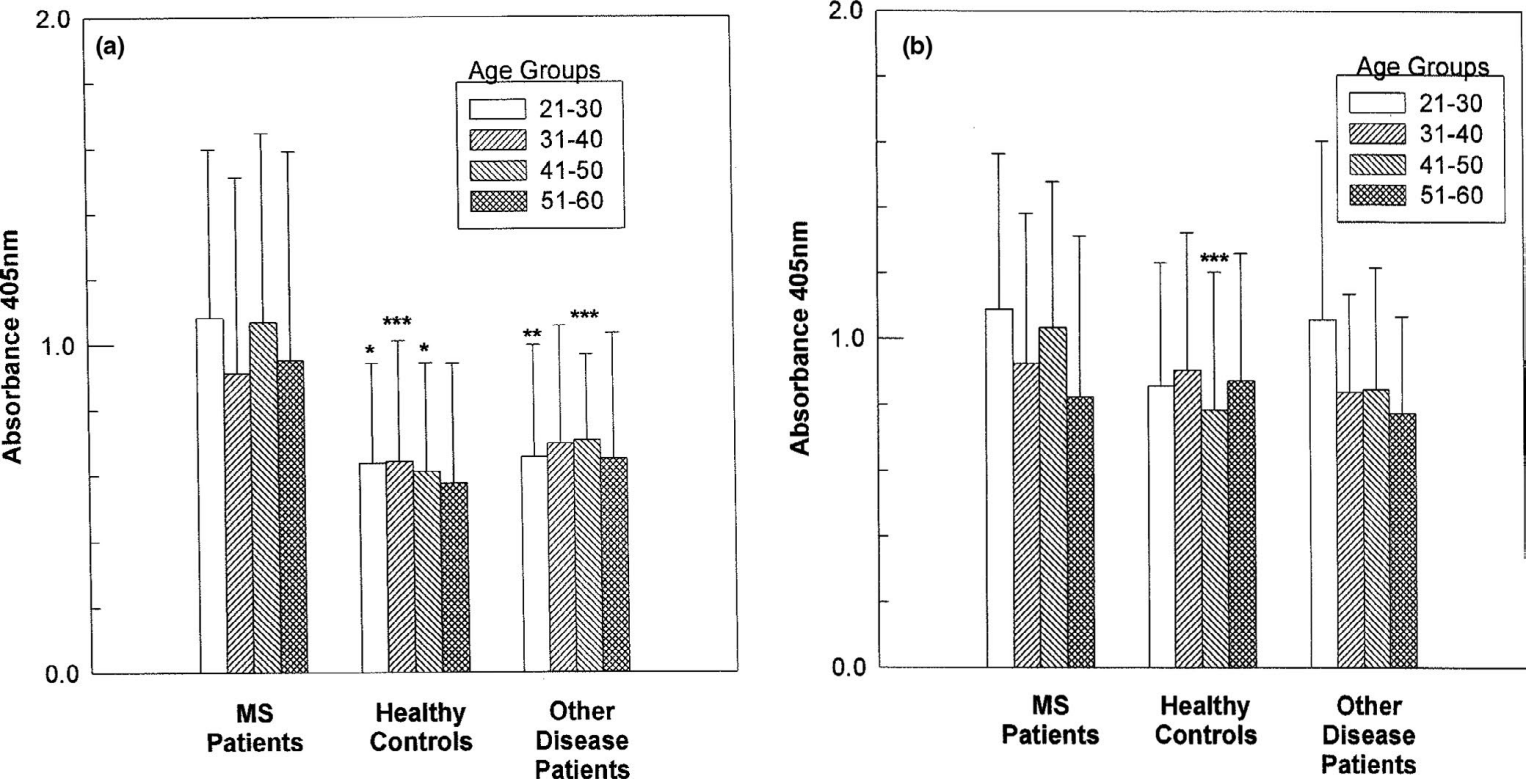
Frequency of CDV‐Hl and MV‐H2 antibodies in patients and controls at different ages. MS and OD patients and HC were divided into groups consisting of 10‐year age intervals. Absorbance values of CDV‐Hl (a) and MV‐H2 (b) IgG are shown for MS patients and controls divided into four age groups. The number of patients (*n*) tested in each (age group) is as follows: For CDV‐H1, (21–30): MS, *n* = 32, HC, *n* = 37, OD, *n* = 23; (31–40): MS, *n* = 47, HC, *n* = 48, OD, *n* = 31; (41–50): MS, *n* = 45, HC, *n* = 31, OD, *n* = 33; and (51–60): MS, *n* = 17, HC, *n* = 12, OD, *n* = 34. For MV‐H2, (21–30): MS, *n* = 29, HC, *n* = 35, OD, *n* = 29; (31–40): MS, *n* = 46, HC, *n* = 45, OD, *n* = 25; (41–50): MS, *n* = 44, HC, *n* = 28, OD, *n* = 28; and (51–60): MS, *n* = 16, HC, *n* = 11, OD, *n* = 28. Results are expressed as the mean ± *SD*. * = *p* < .0001; ** = *p* < .001; *** = *p* < .05

For the analysis of CDV‐Hl and MV‐H2 Abs at different ages, the MS and OD patients and HC were divided into 10‐year age interval groups. Anti‐CDV‐H levels remained stable with little change over a wide age span (Figure 2a). Analysis of MS patients aged 11–20 showed a tendency for elevated anti‐CDV‐H levels although the numbers were too small to reach significance (MS and HC, *n* = 4, and OD, *n* = 3). MS patients exhibited significantly higher levels of anti‐CDV‐Hl than HC (*p* < .05) and OD patients (*p* < .001) in the 21‐ to 30‐year‐old group (Figure 2a). In the 31‐ to 40‐year‐old group, anti‐CDV‐Hl levels were significantly elevated (*p* < .05) in the MS patients compared with the HC and not quite significant in comparison with the OD patients. In the 41‐ to 50‐year‐old group, the levels of CDV­H1 were significantly higher in the MS patients compared with the HC (*p* < .0001) and with the OD patients (*p* < .05). MS patients only had elevated anti‐MV‐H2 levels compared with HC (*p* < .05) in the 41‐ to 50‐year‐old group (Figure 2b). For the 51‐ to 60‐year‐old group, there was a trend for anti‐CDV‐H1 to be higher in MS patients compared with both control groups. Levels of anti‐CDV‐H2 and anti‐CDV‐H3 also remained stable and elevated over the wide age span (data not shown).

### 3.6 Total IgG levels in sera

There was no significant difference in the IgG content between MS (11.4 ± 5.3 mg/ml) and OND patients (12.6 ± 10.0 mg/ml) or HC (13.7 ± 4.7 mg/ml). Comparison of IgG levels in individuals who had CDV‐H Abs and those who were negative for CDV Abs did not show any significant differences in serum IgG levels in MS patients or the controls (data not shown).

### 3.7 Analysis of binding of purified IgG

**FIGURE 3:**
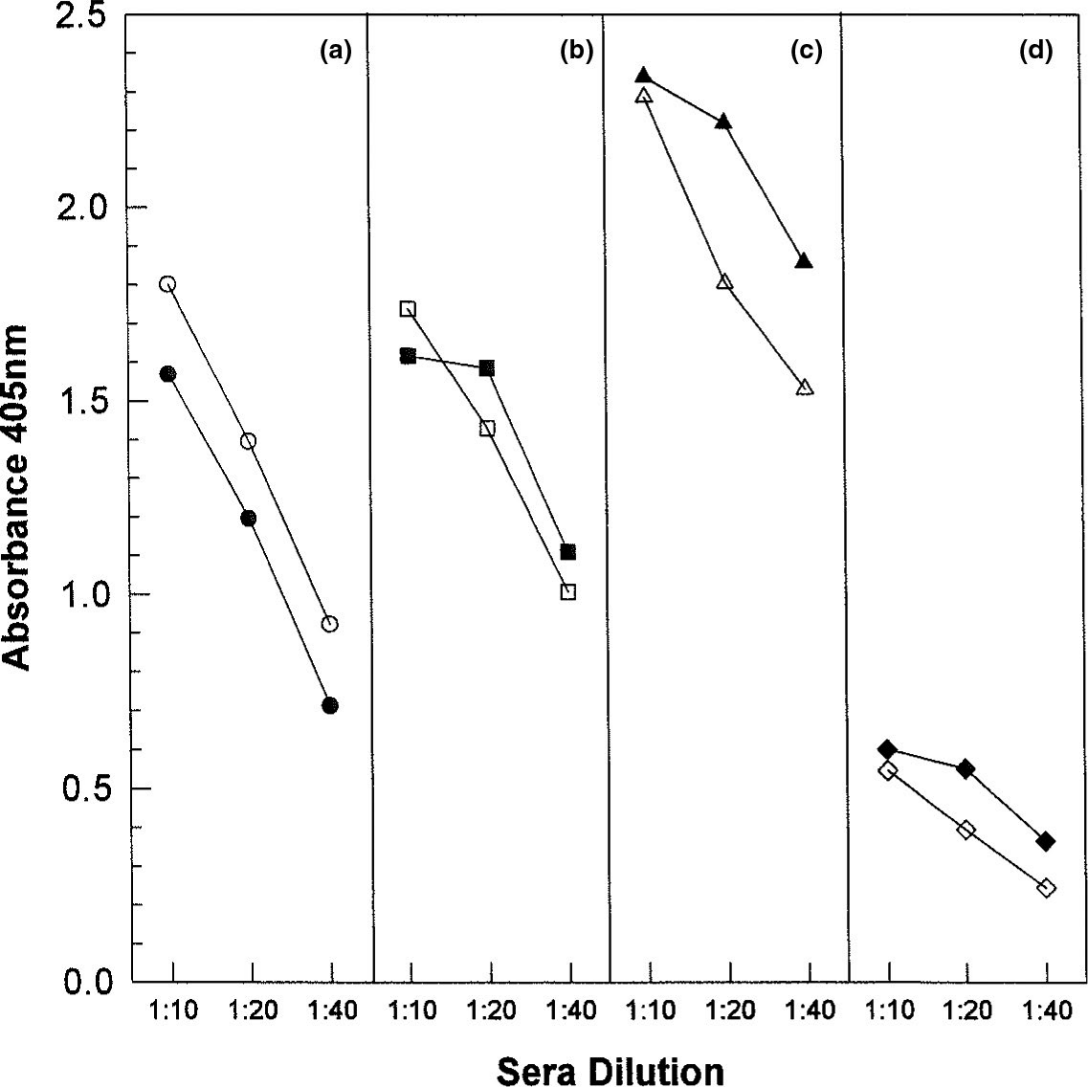
CDV‐H IgG levels in sera and purified IgG samples. Binding of serial dilutions of purified IgG samples and the corresponding sera to CDV‐H761 peptide were measured by ELISA. Results are expressed as mean absorbance at 405 nm for triplicate wells. Absorbance values for four representative individuals (a, b, c, and d) are shown. For each individual, the purified IgG sample is depicted by closed circles, while sera sample is shown by open circle

To further show that we were measuring binding of IgG‐specific antibodies, we purified IgG from the sera of several MS patients and HC. Three sera contained anti‐CDV­H1, while three sera were negative for anti‐CDV‐H1. Serial dilution of sera and the corresponding purified IgG sample showed comparable absorbance values for binding to CDV­H1 peptide in ELISA (Figure 3).

## 4 DISCUSSION

In the present study, we showed that anti‐CDV‐H levels were significantly higher in each decade from 20 to 50 years of age in MS patients compared with healthy and disease controls. Levels of MV Abs were elevated only in the 41‐ to 50‐year‐old group. We confirmed and extended the previous study by measuring anti‐CDV levels in a cohort of patients with IAD and by determining antibody levels to a homologous peptide from the MV‐H protein and to VZV. We demonstrated that CDV‐H Abs levels are significantly increased in MS patients compared with the AID patient group, indicating that such Abs levels are not a secondary consequence of an inflammatory reaction. An increase in anti‐MV levels was found in MS patients compared with healthy and OND controls but not with IAD patients. No difference in anti‐VZV levels was found between MS patients and controls.

There are several notable features of both studies. The randomly selected MS patient and control populations had reasonably similar demographic characteristics. The two studies were performed independently of each other and by different laboratory personnel, with similar results. Assessment of the sera with high levels of anti‐CDV‐H demonstrated that 70% and 61% were from MS patients in the original and present study, respectively. The striking similarities in the odds ratio obtained in the separate and combined analysis of the two studies provide compelling evidence for a strong association between elevated CDV‐H Abs and MS. To the best of our knowledge, the increased risk of MS (OR >5) when antibodies to all three CDV peptides are elevated and an even greater risk (OR >7) when antibodies to CDV‐H1 are elevated is greater than for any genetic or environmental factor previously described. Most important, this risk is independent of the effect of the measles virus. Thus, analyses performed on two large independent cohorts of MS patients, patients with various diseases, and HC demonstrate that anti‐CDV‐H is detectable with the highest levels in MS patients. These studies support the hypothesis that there is an association between antibodies to this neurotropic dog virus and MS. Further studies are needed to determine whether this virus may trigger MS in a subset of individuals.

Increased B‐cell immune reactivity to a variety of viruses has been described in MS (Tselis, 2011). Consistent with published studies, we observed elevated MV Abs in MS patients compared to patients with OND and HC (Adams & Imagawa, 1962; Brody et al., 1972; Haire et al., 1973). However, we found similar anti‐MV levels in MS and AID patients, suggesting that such Abs may be a secondary consequence of an inflammatory response. The elevated anti‐MV‐H2 in MS patients compared with HC is only seen in the 41‐ to 50‐age group and not in the earlier age decades where one would predict increased antibody levels to a virus possibly associated with triggering MS. Several lines of evidence indicate that the elevated anti‐CDV‐H levels are not due to a hyperactive immune system. First, no significant difference between MS patients and HC was observed in the levels of anti‐VZV. Second, CDV‐H1 reactive and CDV‐Hl nonreactive patients and controls showed no difference in anti‐VZV levels. Third, we detected the highest antibody levels to CDV and to a much lesser extent to MV. Moreover, the odds ratio of 7.0 or the presence of elevated anti‐CDV‐H1 and MS diagnosis was more than doubled that for the presence of elevated measles Abs and MS diagnosis. Lastly, no differences in serum IgG levels were observed between MS patients and controls regardless of the presence or absence of CDV‐H Abs. CDV‐H antibody levels do not appear to be affected by immunomodulatory agents such as steroids or interferon‐β (Betaseron®). We observed no significant difference in absorbance values when patients on these therapies were included or excluded from the analysis (Rohowsky‐Kochan et al., 1995).

Exposure to animals, specifically dogs, may be linked with an increased risk of MS, although conflicting results have been reported in support of this association (Anderson et al., 1984; Bansil et al., 1997; Bauer & Wikstrom, 1977; Cook & Dowling, 1977; Cook et al., 1978; Flodin et al., 1988; Hernán et al., 1999; Hughes et al., 1980; Jotkowitz, 1977; Landtblom et al., 1993; Mititelu et al., 1986; Norman et al., 1983; Read et al., 1982; Siejka et al., 2016; Warren et al., 1982). Significantly more exposure of MS patients than controls to dogs with a CDV‐like illness has been reported (Anderson et al., 1984; Cook, et al., 1978; Warren et al., 1982), and others noted a trend that did not reach statistical significance toward more exposure to dogs with a CDV‐like illness in MS patients (Bauer & Wikstrom, 1977; Hughes et al., 1980; Read et al., 1982). A recent study showed that exposure to dogs in early adolescence was associated with an increased risk of MS (Jong et al., 2019). In our studies, we collected and analyzed sera from patients and controls without information regarding dog exposure to avoid sample biasing. We have preliminary data regarding dog exposure of both MS patients and HC from our original study as assessed by a family history questionnaire. With respect to dog ownership, 80% of MS patients had dogs prior to disease onset compared with 55% of HC (unpublished observations). Forty‐three percent of MS patients were exposed to a dog with distemper or a distemper‐like illness compared with 6% of HC (*p* = .01, Fisher's exact test, odds ratio = 13.1). The possibility that a bias existed in answering the survey cannot be ruled out, although exclusion of MS patients and controls who were exposed to a distempered dog did not substantially change mean CDV‐H levels for MS patients (1.024 ± 0.54) and controls (0.612 ± 0.32).

Molecular mimicry between a virus and brain antigen may trigger an autoimmune response resulting in demyelination (Fujinami & Oldstone, 1985). A search through the Swiss‐Prot database revealed that all 3 CDV‐H peptides have a very limited (3–4 amino acids) match with myelin‐associated glycoprotein (MAG and CDV‐H1; MAG and CDV‐H3), myelin oligodendrocyte glycoprotein (MOG and CDV‐H3), and proteolipid protein (PLP and CDV‐H2). The significance of this limited homology is not known. Whether CDV Abs are a result of a secondary epiphenomenon due to myelin breakdown is unlikely since elevated anti‐CDV‐H levels were not found in patients with neurological diseases in which myelin breakdown occurs.

The role of viral infections in MS is complex and still needs to be defined. We provide plausible, significant serological evidence corroborating epidemiologic data suggesting that CDV may be associated with MS, in at least some MS patients. An increased risk of MS (OR >5) was observed when antibodies to all 3 CDV peptides are elevated, and an even greater risk (OR >7) was seen when antibodies to CDV‐H1 are elevated. In comparison, studies linking antibodies to EBV nuclear antigen (EBNA) and the risk of MS observed relative risks ranging from 2.1 to 3.9, in individuals with a fourfold increase in Ab titers (Ascherio et al., 2001; DeLorenze et al., 2006), whereas the risk of 3.2 and 5.1 was seen in individuals with high titers (1:320 and 1:640, respectively) of anti‐EBNA (Levin et al., 2005). In a longitudinal follow‐up, a fourfold increase in anti‐EBNA titers was associated with a risk of 3.0 (Levin et al., 2005). Increased EBNA‐specific IgG responses predicted conversion to MS in CIS patients with an OD = 2.2 (Lunemann et al., 2010). We emphasize, however, that our studies do not prove that CDV causes MS; neither CDV genome nor antigen have been identified in MS tissues, not all individuals with high CDV antibody titers have MS and some MS patients have low CDV antibody levels. Additional molecular and serological studies are warranted to confirm this hypothesis. As MS is a heterogeneous disease, the possibility that more than one virus may be involved in the pathogenesis and the interaction between viruses and other infectious, genetic, or environmental factors should be considered.

## 5 CONCLUSION

The results of this study provide plausible, significant serological evidence corroborating epidemiologic data suggesting that CDV may be associated with MS, in at least some MS patients. Individuals with antibodies to the three CDV peptides have a five times greater chance of having MS than individuals in whom such antibodies were not detected and the risk of MS is independent of the effect of measles. The finding that anti‐CDV levels are elevated in MS patients of all ages provides substantial evidence of a strong association between elevated anti‐CDV and MS.

## CONFLICT OF INTEREST

The authors declare no financial or other conflicts of interests.

## AUTHOR CONTRIBUTION

Dr. Rohowsky‐Kochan conceived the idea for this paper, designed all the experiments, analyzed the data, and wrote the original and revised manuscript. Dr. Amy Davidow was instrumental in providing statistical analysis needed for the revised manuscript. Dr. Peter Dowling contributed to providing the sera samples from patients with other neurological diseases as well as from patients with autoimmune, inflammatory diseases. He was involved in the critical reading of the manuscript. Dr. Stuart Cook was instrumental in recruiting the MS patients and some of the other neurological disease patients to the study. He provided the clinical data on the patients. Dr. Cook was actively involved in reviewing the manuscript and providing helpful suggestions.

### Peer Review

The peer review history for this article is available at https://publons.com/publon/10.1002/brb3.1920.

## Data Availability

The data that support the findings of this study are available from the corresponding author upon reasonable request.
